# Essential role of the initial activation signal in isotype selection upon deletion of a transcriptionally committed promoter

**DOI:** 10.1038/s41598-019-54929-x

**Published:** 2019-12-06

**Authors:** Joana M. Santos, Chloé Oudinet, Lisa Schöne, Audrey Dauba, Ahmed Amine Khamlichi

**Affiliations:** Institut de Pharmacologie et de Biologie Structurale, Université de Toulouse, CNRS, Université Paul Sabatier, 31077 Toulouse, France

**Keywords:** Class switch recombination, Epigenetics in immune cells

## Abstract

Class switch recombination (CSR), which targets exclusively the constant region of the immunoglobulin heavy chain (*IgH*) locus, plays an important role in humoral immunity by generating different antibody effector functions. The *IgH* constant locus contains multiple genes controlled by isotype (I) promoters induced by extracellular signals that activate specific I promoters, leading to B cell commitment. However, it is unknown whether after initial commitment to one promoter, non-responsive I promoters are irreversibly silent or if they can be activated after exposure to their specific inducers. Here, we studied the murine cell line CH12, which is committed to produce IgA in response to TGF-β. We show that, although other promoters than Iα are transcriptionally inactive, they are not irreversibly silent. Following deletion of the committed Iα promoter by CRISPR/Cas9, other I promoters display a complex transcriptional pattern largely dependent on the initial committing signal.

## Introduction

Humoral immunity relies on a vast repertoire of B cell antigen receptors generated through different processes along B cell development^1,2^. At the immunoglobulin heavy chain (*IgH*) locus, the variable region undergoes V(D)J recombination in developing B cells leading to the assembly of the variable gene segments (V(D)J)^3–5^. The constant region undergoes class switch recombination (CSR), enabling IgM-expressing B cells to switch to other isotypes (IgG, IgE, IgA)^6–8^.

The *IgH* constant region contains multiple constant (*C*_*H*_) genes whose transcription initiates at isotype-specific promoters, called I promoters^6^. The I promoters are largely controlled by the super-enhancer 3′RR (3′ Regulatory Region), composed of four enhancers (hs3a, hs1-2, hs3b and hs4), located downstream of the *IgH* locus^9^.

Transcription from I promoters is induced upon antigen encounter and signaling from other immune cell types^6^. I-derived transcription elongates across highly repetitive sequences, called switch (S) sequences, and generates secondary structures facilitating recruitment of the enzyme AID (*e*.*g*.^10–12^). AID initiates DNA cleavage at the universal donor Sµ region and the activated downstream S region. Ligation of the two S regions brings the downstream constant region into proximity of the rearranged VDJ gene segment, ultimately leading to the expression of a new isotype^6,7^.

Seminal studies showed that B cells activated by a given signal are transcriptionally committed towards the activated isotype(s) prior to recombination to that particular isotype(s)^13–15^. This pre-switch “transcriptional commitment” model has since been confirmed by various mutational studies targeting I promoters (*e*.*g*.^16–19^). However, it is unknown whether initially non-activated I promoters are irreversibly silent or if they can be activated when committed B cells are subsequently exposed to inducers promoting their activation.

By using the IgA-committed B cell line CH12, we show that non-committed I promoters are not irreversibly silent. Following deletion of the committed Iα promoter, activation of the I promoter responding to the initial activating cytokine is favored.

## Results

### CRISPR/Cas9-mediated deletion of the Iα promoter in the CH12F3-2 cell line

The murine cell line CH12 is derived from the CH12.LX lymphoma cell line. This cell line is transcriptionally committed to the Iα promoter, which has basal activity even in the absence of stimulation, and activated CH12 cells switch exclusively to IgA^20,21^. Throughout this study, we used the sub-clone CH12F3-2^22^ (hereafter called CH12 line or cells).

**Figure 1:**
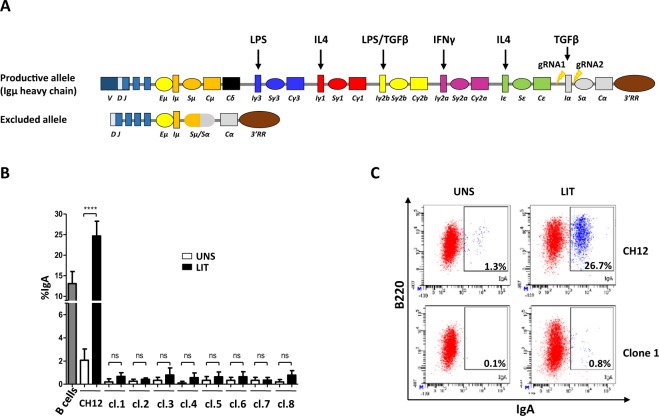
Deletion of Iα promoter/exon inhibits CSR to IgA. (**A**) Schematic structure of the *IgH* locus in CH12F3-2 line. The non-expressed allele is a partially rearranged DJ_H_ allele that underwent Sµ/Sα recombination, thus deleting all upstream inducible I promoters. The mitogen and cytokines inducing the different I promoters are indicated on top. The sites flanking Iα promoter/exon targeted by the gRNAs are indicated with arrows. The Eµ/Iµ enhancer/promoter between the variable and the constant regions, and the 3′RR super-enhancer downstream of the locus are shown. (**B**) Flow cytometry analysis of Iα-deleted clones. The 8 clones obtained by CRIPR/Cas9 were analyzed by FACS for IgA surface expression. The parental CH12 line was used as a control prior to (UNS) and following LIT (LPS + IL4 + TGFβ) stimulation. LIT-activated splenic B cells were also included as a control (n = 3). (**C**) Representative FACS plot obtained with CH12 cells and an Iα-deleted clone (n° 1), before and following LIT stimulation.

To investigate the effect of deleting the committed Iα promoter on activation of upstream I promoters and CSR, we designed two CRISPR/Cas9 guide RNAs specifically targeting the Iα promoter/exon (Fig. 1A). Because in CH12, the non-productive allele has already undergone Sµ/Sα recombination^22,23^ (Supplementary Fig. S1A), which deleted all I promoters except for the Eµ/Iµ enhancer/promoter (Fig. 1A), the gRNAs target exclusively the productive allele. PCR screening and sequencing identified eight clones with the desired deletion (Supplementary Fig. S1A–D).

FACS analyses showed that in response to LIT (LPS + IL4 + TGFβ), CH12 cells undergo robust CSR to IgA to levels higher than in activated splenic B cells (Fig. 1B). As expected, none of the Iα-deleted clones switches to IgA (Fig. 1B,C). We checked on three random mutant clones that no *trans*-splicing occurred between the VDJ exon of the productive allele and the Cα region of the non-productive allele (Supplementary Fig. S1E).

### Switch transcription and CSR in activated CH12 cells and deletion clones

To determine if CSR occurs in the absence of the committed Iα promoter, we first tested switching under stimulation conditions known to induce switching in primary B cells; LPS stimulation induces CSR to IgG3 and IgG2b, LPS + IL4 to IgG1 and IgE, LPS + IFNγ to IgG2a, and LIT to IgG2b and IgA.

**Figure 2:**
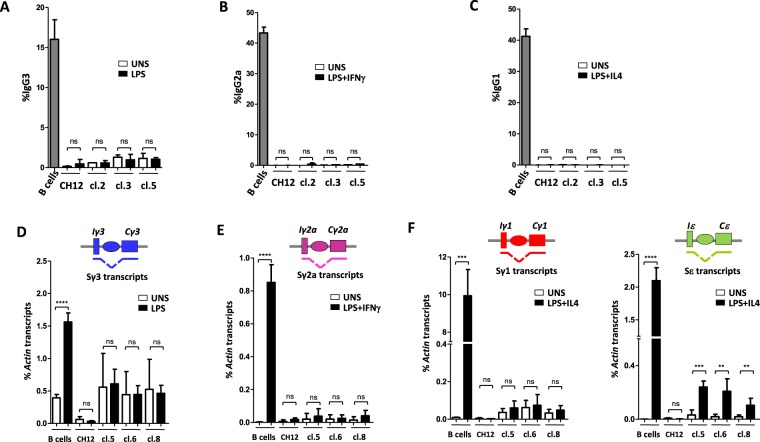
Iα-deleted clones fail to undergo CSR following specific stimulation. (**A**–**C**) CH12 cells, three Iα-deleted clones, and splenic B cells were activated by LPS (**A)**, LPS + IFNγ (**B**) or LPS + IL4 (**C**) and stained for IgG3, IgG2a and IgG1, respectively. Representative plots are shown for unstimulated (UNS) and activated CH12 cells, Iα-deleted clones (clone 5) and primary B cells. **(D**–**F)** RT-qPCR quantification of pre-switch transcripts (Sx transcripts) in unstimulated and day 2 activated splenic B cells, CH12 cells or clones 5, 6 and 8 in response to LPS (Sγ3) (**D**), to LPS + IFNγ (Sγ2a) (**E**), or to LPS + IL4 (Sγ1 and Sε) (**F**) (n = 3).

FACS analysis revealed that, in contrast to activated splenic B cells, in CH12 as well as in all Iα-deleted clones, LPS, LPS + IFNγ and LPS + IL4 failed to induce CSR to IgG3, IgG2a and IgG1, respectively. None of these stimuli induced CSR to IgA, as expected (Fig. 2A–C, Supplementary Figs. S2 and S3). These results were confirmed in three random clones by RT-qPCR quantification of post-switch transcripts^24^ (Supplementary Fig. S4).

Quantification of pre-switch transcripts in unstimulated (UNS) cells revealed that transcripts levels were higher in deletion clones than in CH12, except for Sγ2a (Fig. 2D–F, Supplementary Fig. S5). Moreover, with the exception of Sε transcripts, whose levels increased in activated Iα-deleted clones (Fig. 2F), switch transcripts were not further induced following stimulation (Fig. 2D–F).

We conclude that deletion of the committed Iα promoter up-regulates most non-committed I promoters.

### The Iγ2b promoter is induced in response to LIT but not LPS stimulation in CH12

In primary B cells, CSR to IgG2b is induced in response to LPS or LIT. We asked if CSR to IgG2b in CH12 cells is induced by either stimulus or only in response to the inducer of the committed isotype (*i*.*e*. LIT).

**Figure 3:**
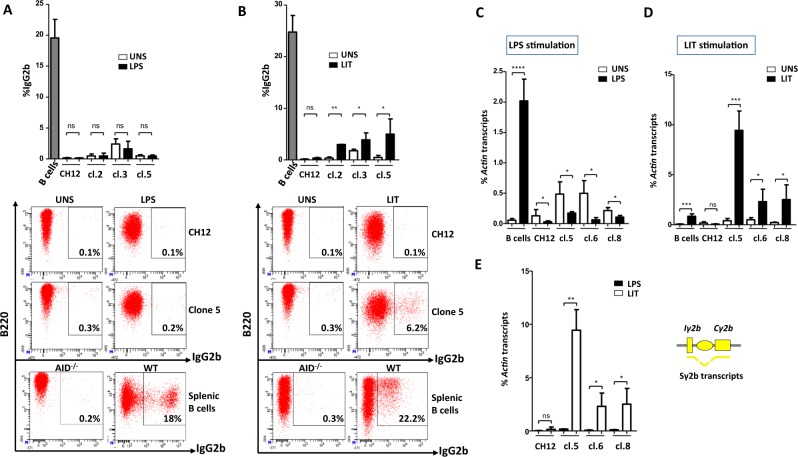
CSR to IgG2b is partially restored in response to TGF-β but not to LPS stimulation. (**A**,**B**) CH12 cells, three Iα-deleted clones (2, 3, and 5) and splenic B cells were activated by LPS (**A**) or LIT (**B**) for 4 days, and stained for IgG2b. Representative plots are shown for unstimulated (UNS), activated CH12 cells, Iα-deleted clones (clone 5) and primary B cells. (**C**,**D**) RT-qPCR quantification of Sγ2b pre-switch transcripts levels in unstimulated clones 5, 6 and 8 and in response to LPS (**C**) or to LIT (**D**) (day 2). (**E**) Comparison of Sγ2b pre-switch transcripts levels in Iα-deleted clones 5, 6 and 8, following LPS and LIT stimulation (n = 3).

FACS analysis revealed that, unlike primary B cells, LPS stimulation did not induce CSR to IgG2b in either CH12 or Iα-deleted clones (Fig. 3A). While in response to LIT, CH12 cells also failed to switch to IgG2b, Iα-deleted cells underwent significant switching to IgG2b (Fig. 3B). Although they varied from clone to clone, the levels of CSR to IgG2b in mutant clones were always lower compared to CSR to IgA in CH12 cells (Figs. 1B,C and 3B). Accordingly, Iµ-Cγ2b transcripts levels increased following LIT stimulation only (Supplementary Fig. S6). Moreover, higher levels of Sγ2b pre-switch transcripts were detected in LIT-stimulated clones compared to LPS-stimulated counterparts (Fig. 3C–E). Surprisingly, unlike primary B cells where LPS induces Sγ2b transcription, LPS repressed Sγ2b transcription in CH12 line as well as in all Iα-deleted clones (Fig. 3C).

This data shows that Sγ2b transcription and subsequent CSR to IgG2b are induced in Iα-deleted clones, but only in response to the inducer of the committed isotype (LIT).

### Differential induction of switch transcription and CSR in the presence of TGF-β

The unexpected finding that IgG2b only responds to LIT stimulation suggested to us that the Iγ2b promoter responds differently in the CH12 line versus primary B cells. Given that high switching levels to IgA can be achieved when activating CH12 cells with anti-CD40 + IL4 + TGFβ (CIT)^21,22^, we wondered if and how this stimulus would impact CSR to IgG2b.

**Figure 4:**
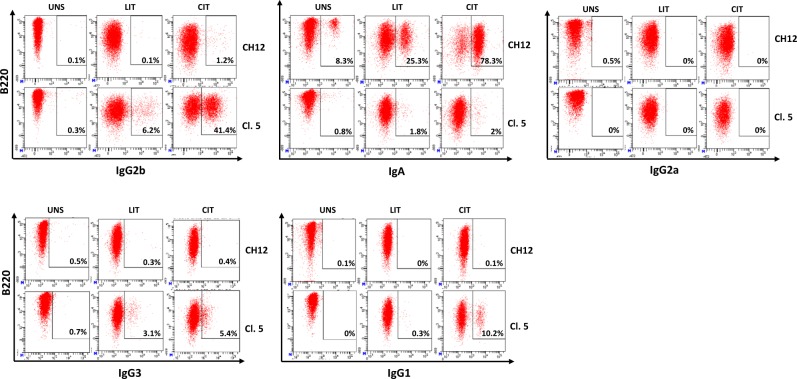
Differential induction of CSR in response to LIT and CIT. CH12 cells and three Iα-deleted clones (3, 5 and 8) were activated by LIT or CIT for 4 days, and stained for the indicated isotypes. Representative plots are shown for activated CH12 cells and Iα-deleted clones (clone 5) (n ≥ 3).

In CH12, cells switched at higher levels to IgA under CIT than LIT (Figs. 1B and 4) and a low percentage of cells switched to IgG2b in the presence of CIT (Figs. 3B and 4). Similarly, In Iα-deleted clones, switching to IgG2b was considerably higher with CIT than with LIT (Fig. 4). Given the effect of CIT on CSR to IgG2b, we analyzed CSR to other isotypes in CIT-treated cells. We found that switching to IgG3 occurred at varying efficiencies but that switching levels were higher in response to CIT than to LIT (Fig. 4). Switching to IgG1 occurred only with CIT while CSR to IgG2a was undetectable regardless of the stimulation (Fig. 4).

**Figure 5:**
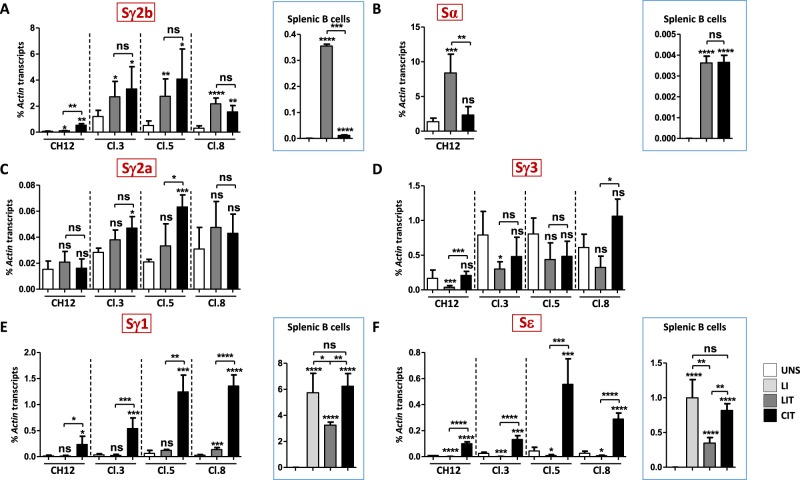
Differential induction of switch transcription in response to LIT and CIT. (**A**–**F**) RT-qPCR quantification of Sγ2b (**A**), Sα (**B**), Sγ2a (**C**), Sγ3 (**D**), Sγ1 (**E**), and Sε (**F**) pre-switch transcripts levels in CH12 cells and Iα-deleted clones 3, 5 and 8 following LIT or CIT stimulation. Transcripts levels in splenic B cells activated with LIT and CIT for Sγ2b (**A**) and Sα (**B**), and with LPS + IL4 (LI), LIT and CIT for Sγ1 and Sε (**E**,**F**) are boxed (n ≥ 3).

We then asked if the increment in switching with CIT, as compared to LIT, was accompanied by an increase in switch transcription. While Sγ2b pre-switch transcripts levels increased upon CIT stimulation in CH12 cells, there were no differences in transcript levels between LIT and CIT in deletion clones, whereas in splenic B cells, Sγ2b transcripts levels were higher with LIT (Fig. 5A). Surprisingly, while FACS detected higher CSR to IgA with CIT, Sα transcripts levels in CH12 cells were higher with LIT than with CIT, but there was no difference in activated splenic B cells (Fig. 5B). Iα-deleted clones, as expected, did not produce Sα transcripts.

Although there was increased CSR to IgG3 with CIT and no switching to IgG2a, regardless of stimulation, Sγ2a (with the exception of clone 5) and Sγ3 transcripts levels were comparable between LIT- and CIT-activated deletion clones (Fig. 5C,D).

In CH12 cells and in deletion clones, LIT repressed Sε, but not Sγ1 transcription, except for clone 8 in which Sγ1 transcripts increased. CIT induced Sγ1 and Sε transcription (Fig. 5E,F and Supplementary Fig. S7). In splenic B cells, CIT was just as efficient as LPS + IL4 (LI) in inducing Sγ1 and Sε transcription, and the levels were higher than with LIT (Fig. 5E,F).

Thus, in the absence of the committed Iα promoter, CSR to IgG2a does not take place, and while switching to IgG1 occurs with CIT only, switching to IgG2b and IgG3 occurs in response to both CIT and LIT. However, switching was constantly higher in response to CIT. Surprisingly, switching efficiency did not always correlate with switch transcripts levels.

### Lack of correlation between *Aicda* transcripts levels and CSR efficiency

Since the enzyme AID is absolutely required to initiate CSR, we wondered if the lack of correlation between switch transcription and CSR in some cases is due to lower expression of the *Aicda* gene, encoding AID.

**Figure 6:**
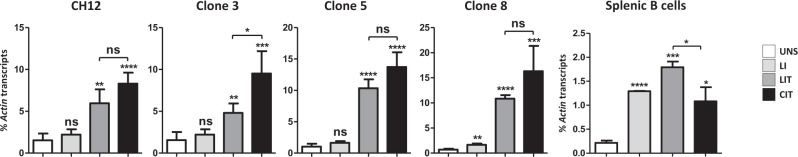
Stimulus-dependent induction of *Aicda* gene transcription. CH12 cells, three Iα-deleted clones (3, 5 and 8) and splenic B cells were activated with LPS + IL4 (LI), LIT or CIT for 2 days. Total RNAs were collected from unstimulated and activated cells and *Aicda* transcripts were quantified by RT-qPCR. *Actin* transcripts were used for normalization (n ≥ 3).

We found that *Aicda* transcripts levels were higher in CH12 cells and derived clones compared to primary B cells, both in unstimulated and stimulated conditions (Fig. 6). LI (LPS + IL4) did not induce *Aicda* transcription in CH12 and Iα-deleted clones, but only in primary B cells (Fig. 6). In contrast to LI, LIT and CIT efficiently induced *Aicda* gene in all cells (Fig. 6).

Therefore, while absence of switching to Sε in CH12 and deletion clones following LI stimulation correlates with the lack of induction of *Aicda* gene, the same is not true for the differences in CSR to IgG2b and IgA in the presence of LIT and CIT.

### Increased transcription of hs1-2 enhancer in response to LIT but not CIT

Enhancer RNAs (eRNAs) are produced at the 3′RR following activation of splenic B cells and are a hallmark of 3′RR activity^25–27^. We then wondered if the non-correlation between switch transcription and CSR, in the cases where there is similar activation of *Aicda*, could be explained by differences in the transcriptional activity of the 3′RR. In order to test this, we quantified hs3a, hs1-2 and hs3b transcripts levels.

**Figure 7:**
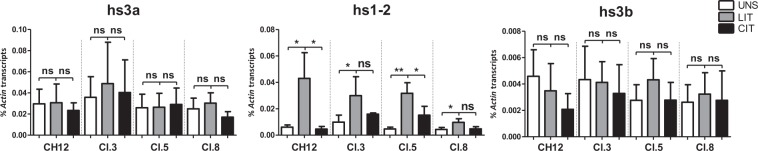
3′RR transcripts levels upon LIT and CIT stimulations. RT-qPCR quantification of eRNAs levels of hs3a, hs1-2 and hs3b enhancers. Total RNAs were extracted from unstimulated CH12 cells and Iα-deleted clones (3, 5, and 8) at day 2 post-stimulation. *Actin* transcripts were used for normalization, and (-RT) controls were included throughout (n ≥ 3).

eRNAs levels were comparable between unstimulated CH12 cells and deletion clones, and were higher than in splenic B cells (Fig. 7). For both CH12 cells and deletion clones, while hs3a and hs3b eRNAs levels did not significantly vary with LIT and CIT (despite some clonal variation) (Fig. 7), there was a consistent trend towards increased hs1-2 transcription with LIT (Fig. 7).

While enhanced transcription of hs1-2 in response to LIT correlates with low levels of Sγ1 and Sε transcripts, high levels of Sγ2b and Sα transcription were detected in the presence of LIT. Therefore, increased transcription of hs1-2 enhancer cannot alone explain the dissociation between switch transcription and CSR.

## Discussion

The CH12 cell line is widely used to study various aspects of CSR (*e*.*g*.^11,12,22,23,28^). However, transcriptional activity of its I promoters had not yet been studied. We investigated the transcriptional status of the non-committed I promoters and CSR levels in the presence or absence of the initially committed Iα promoter, following various stimulations classically used for primary B cells and/or CH12.

Treatment of the parental CH12 line with LPS, LI or LPS + IFNγ did not activate any of the I promoters. However, the block was not irreversible because, upon deletion of the committed Iα promoter, switch transcripts levels of all isotypes (with the possible exception of Sγ2a) increased in unstimulated cells. This indicates that simple deletion of the committed promoter enabled other I promoters to acquire a relatively open chromatin state.

Upon stimulation of Iα-deleted clones, the I promoters displayed different responses. For instance, while Iγ1 was induced by CIT only, Iε was induced by LI and, more strongly, by CIT, but was repressed by LIT. Because both LIT and CIT contain TGF-β, strong induction of Iε and of Iγ1 only in the presence of CIT was surprising. It has been shown that, in splenic B cells, TGFβ induces the Id2 repressor, which antagonizes binding of basic helix-loop-helix E2A and PAX5 transcription factors, precluding activation of the Iε promoter (E2A in the case of Iγ1)^29^. We found that Sγ1 and Sε transcripts levels were reduced in splenic B cells activated with LIT, compared to CIT. Together, these findings indicate that TGFβ impacts Sγ1 and Sε transcription in the presence of LPS, but not of anti-CD40. This suggests that signaling through CD40 somehow circumvents TGFβ-induced Id2-mediated suppression of transcription factors activity. The Toll-like receptor 4 (TLR4, which binds LPS) and CD40 preferentially trigger the classical and the alternative NF-κB signaling pathways, respectively^30,31^. Nonetheless, LPS can activate both pathways through binding to both TLR4 and surface IgM^32,33^. Given that all I promoters (except Iµ) and the 3′RR have NF-κB binding sites^6,9,34^, this suggests that the cross-talks between TGFβ and LPS pathways on one hand, and TGFβ and CD40 ligand pathways on the other hand, have more complex transcriptional outcomes than previously thought.

CIT induced higher levels of CSR than LIT, but there was not always a correlation between increased CSR and switch transcription as illustrated by increased Sα transcription in CH12 cells in the presence of LIT, and similar Sγ2b transcript levels in deletion clones under the two stimulations. This suggested that other mechanisms might be involved. We investigated transcription of *Aicda* gene under different stimulations. We showed that LIT and CIT, but not LI, efficiently induced *Aicda* in CH12 cells and deletion clones. Nonetheless, there was not always a correlation between switching efficiency and *Aicda* induction. On one hand, the failure to switch to Sε in response to LI could be due to non-induction of *Aicda*, but also to low levels of Sε transcripts, or to a conjunction of both. On the other hand, CIT induced higher levels of CSR to IgA in CH12 cells, and to IgG2b in deletion clones, than LIT but this did not correlate with a substantial increase in *Aicda* gene induction. Therefore, AID levels *per se* cannot explain the large difference in switching efficiency in response to CIT *versus* LIT. We suggest that factors acting downstream of AID may be more strongly induced by CIT than LIT.

**Figure 8:**
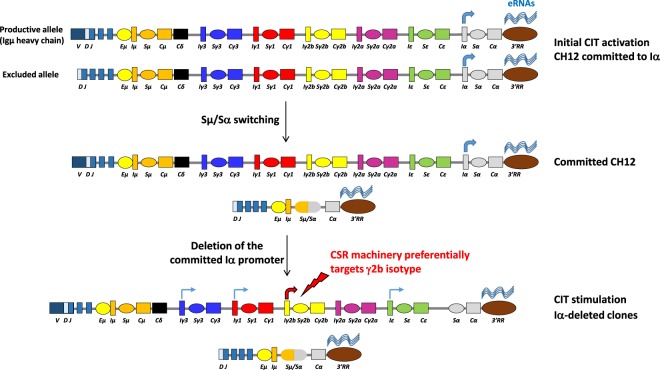
Model of the natural history of the CH12 line. Initial activation of the original B cell clone likely took place in the context of a T-dependent response involving TGF-β and CD40. This led to commitment to Iα promoter and induction of 3′RR transcription on both alleles, and subsequent switching on the non-expressed allele. The CSR machinery retained somehow memory of the initial activating signal (CIT). The Iα promoter and the 3′RR remain active in the committed CH12 line. Upon deletion of the committed Iα promoter, Iγ2b normally induced with either LPS or TGF-β, is only induced in response to TGF-β, but the highest switching levels to IgG2b are preferentially achieved with the initial signal (CIT).

Transcriptional activity of the 3′RR could also account for the complex transcriptional pattern of I promoters. Given that unstimulated CH12 cells and deletion clones expressed similar eRNAs levels, deletion of the committed promoter did not affect 3′RR activity. Additionally, eRNAs levels in CH12 were higher compared to primary B cells, and were insensitive to CIT. This suggests that eRNAs have reached a level sufficient for activation of specific I promoters. In this regard, it should be noted that the CH12 line has already switched on the non-expressed allele, *i*.*e*., it has experienced activation that induced the 3′RR on both alleles (Fig. 8).

Remarkably, hs1-2 transcription could be further induced by LIT, and this correlated with low levels of Sγ1 and Sε transcripts. This pattern is consistent with a model in which Iγ1 and Iε promoters compete with highly transcribed 3′RR enhancers for transcription factors^27^. However, the activation patterns of Iγ2b (similarly induced by LIT and CIT) and Iα (induced by LIT but not by CIT) suggest different or additional mechanisms. Various non-mutually exclusive mechanisms could be involved including local factors specific to I promoters (combinatorial effect of transcription factors, *cis*-acting elements…), preferential interactions of individual 3′RR enhancers with I promoters^35,36^, and physical proximity to the 3′RR. The finding that heightened transcription of hs1-2 enhancer with LIT correlates with reduced CSR to IgG2b and IgA may indicate that hs1-2 enhancer competes with Sγ2b and Sα for AID. Recruitment of AID by super-enhancers has been demonstrated^37,38^ and there is some evidence that AID could target the 3′RR^25^.

A remarkable finding of this study concerns the switch levels to IgG2b in deletion clones that are considerably higher in response to CIT than to LIT. This was unexpected, and as mentioned, cannot be due to higher switch transcripts levels or to 3′RR eRNAs or AID levels, but may suggest that activation of the initial B cell clone (that gave rise to CH12 line) took place in the context of a T-dependent response, which activated the CD40 pathway. Moreover, the CH12 line has already undergone switching on the excluded allele and may therefore represent an advanced state of commitment. Importantly, in the absence of the committed promoter, particularly in the presence of CIT, Sγ2b transcription is more strongly induced than in primary B cells, and, of all isotypes, the highest levels of switching occur towards IgG2b specifically. Thus, in terms of both transcription and switching, the γ2b isotype appears to be the preferential target of CIT, *i*.*e*. the likely initial committing signal. We have recently shown that in the majority of TGFβ-activated splenic B cells, the Iγ2b and Iα promoters compete for the 3′RR^39^. Although we cannot ascertain if commitment to the Iα promoter in the original CH12 line took place following co-activation of Iγ2b and Iα or single activation of Iα, the CSR pattern in deletion clones raises the possibility that commitment to the Iα promoter has rewired the CSR machinery so that, even after deletion of Iα, it targets Iγ2b and there is optimal switching in response to the initial signal (Fig. 8). Whether this coincides with CIT-induced formation of specialized nuclear compartments such as transcription factories^40^ that would facilitate Iγ2b-3′RR interactions and recruitment of AID remains to be explored.

In conclusion, we showed that initial commitment to the Iα promoter in CH12 cells blocks transcriptional activation of other promoters. However, the block is not irreversible. Commitment to a particular isotype appears to target the CSR machinery towards a pathway in which the stimulating cytokine plays a key role so that in the absence of the committed promoter, activation of the I promoter responding to the initial signal is favored. It would be interesting to explore if the same is true for primary B cells.

## Material and Methods

### Cell culture

CH12 and primary splenic B cells (from 129Sv1 mouse strain) were cultured in RPMI media supplemented with 10% heat inactivated serum, 10 mM HEPES, 1 mM sodium pyruvate, 100 U/mL Penicillin, 100 U/mL Streptomycin, 50 μM β-mercaptoethanol, 1x non-essential amino acids.

CH12 cells were stimulated for 2 or 4 days at a density of 10^5^ cells/ml, in the presence of 50 µg/ml of LPS (Sigma) (LPS stimulation); 50 µg/ml LPS and 20 ng/ml IFN-γ (R&D) (IFN-γ stimulation); 50 µg/ml LPS and 25 ng/ml IL4 (eBiosciences) (IL4 stimulation); 50 µg/ml LPS, 10 ng/ml IL4, and 2 ng/ml TGFβ (R&D) (LIT stimulation); 1 µg/ml anti-CD40 (eBiosciences), 10 ng/ml IL4, and 2 ng/ml TGFβ (CIT stimulation). Purification and stimulation of primary splenic B cells were as described (ref. ^40^).

### Molecular cloning

The gRNAs oligonucleotides were phosphorylated with T4 Polynucleotide Kinase (Thermo Scientific) and annealed. Afterwards, they were ligated with B*sa*I or B*bs*I digested pX333 plasmid (Addgene). Confirmed cloning products were used as template to PCR amplify the gRNA cassette with primers gRNA-M*lu*IFw and gRNA-M*lu*IRev, and the PCR fragment was cloned into M*lu*I digested CMV-Cas9-GFP plasmid (Sigma). Correct cloning was diagnosed by restriction digestion and sequencing. All primers are listed in Table S1.

### CH12 cells transfection

2 × 10^6^ cells were transfected with 2 µg of CMV-Cas9-GFP-gRNA by electroporation using program O-006 of the Amaxa Nucleofector II (Lonza) and Amaxa Cell Line Nucleofector kit V. Transfected cells were cultured at 37 °C for 24 h. GFP-positive cells were single-sorted into 96-well plates, cultured for seven days and then PCR tested for the presence of the deletion with the appropriate primers and GoTaq Polymerase (Promega), according to the manufacturer’s instructions. Deletion clones were checked by sequencing.

### RT-qPCR

Total RNAs were collected from non-stimulated or stimulated cells at day 2 or day 4 post-treatment using a commercial kit (Zymo Research). Total RNAs were reverse-transcribed (Invitrogen) and qPCR was performed using Sso Fast Eva Green (BioRad), according to the manufacturer’s instructions. *Actin* transcripts levels were used for normalization and the results are shown as percentage of actin. (-RT) controls were tested for all samples.

### Flow cytometry

At day 4 post-stimulation, cells were washed and stained with anti-B220 APC (BioLegend) and either anti-IgG3-FITC (BD-Pharmingen), anti-IgG1-FITC (BioLegend), anti-IgG2b-PE (BioLegend), anti-IgG2a-PE (BioLegend) or anti-IgA-FITC (BD-Pharmingen). Data were obtained on 3 × 10^4^ viable cells by using a Coulter XL apparatus (Beckman Coulter).

### Statistical analysis

Results are expressed as mean ± SD (GraphPad Prism), and overall differences between values from WT and mutant mice were evaluated by a two-tailed *t* test. The difference between means is significant if *p* < 0.05 (*), very significant if *p* < 0.01 (**), and extremely significant if *p* < 0.001 (***).

## Supplementary information

Supplementary information

## Data Availability

Materials, data and associated protocols are promptly available to readers.
