# Supplementary material for: Essential role of the initial activation signal in isotype selection upon deletion of a transcriptionally committed promoter
Source: Sci Rep. 2019 Dec 6;9:18543. doi: 10.1038/s41598-019-54929-x (PMC6898632; doi:10.1038/s41598-019-54929-x)
Supplement: Supplementary file 1 — Supplementary information [file 41598_2019_54929_MOESM1_ESM.pdf]

Supplementary information

Essential role of the initial activation signal in isotype selection upon deletion of a transcriptionally committed promoter

Joana M. Santos, Chloé Oudinet, Lisa Schöne, Audrey Dauba, Ahmed Amine Khamlichi*

Supplementary figure legends

Figure S1. CRIPR/Cas9-mediated deletion of Iα promoter/exon in CH12F3-2 line

(A) Schematic representation of the productive and excluded alleles in CH12F3-2 line (not to scale). The position of the gRNAs used in this study is shown. The excluded allele already underwent Sμ/Sα recombination. The switch junction (in red) was sequenced and is highlighted below. The asterisks indicate point mutations. **(B)** Scheme of the wild type (WT) and Iα-deleted (ΔIα) locus with the position of the gRNAs and diagnosis primers used, as well as the expected size of the deletion and diagnostic PCRs. **(C)** Diagnosis PCR showing that while in CH12 cells, there is amplification with the primers 6 and 7, only in Iα-deleted clones there is amplification of a PCR product with the primers 133 and 48. Left, scan of the gel; boxed in red and blue are the cropped gel portions of interest highlighted in the middle in the same colors. Right, zoom on the bands of interest with the expected size. There has been no image manipulation. **(D)** Alignment of the sequence amplified with primers 133 and 48 in the different clones; the position of the two gRNAs is highlighted in yellow. Note that the extent of the deletion varied between clones. **(E)** RT-qPCR screening for potential *trans*-splicing. Total RNAs were extracted from three random clones (5, 6 and 8) and subjected to RT-qPCR using a forward primer that pairs with the V_H exon of the productive allele and a reverse primer that pairs with Cα1 exon of both alleles. For clarity, Iμ-Cμ transcripts on the productive allele are not shown.

Figure S2. Iα-deleted clones fail to undergo CSR following specific stimulation

(**A-C**) CH12 cells, three Iα-deleted clones, and splenic B cells were activated by LPS (**A**), LPS+IFNγ (**B**) or LPS+IL4 (**C**) and stained for IgG3, IgG2a and IgG1, respectively.

Representative plots are shown for unstimulated (UNS) and activated CH12 cells, Iα-deleted clones (clone 5 in this example) and primary B cells. AID-deficient B cells (unable to switch) were included as a negative control for primary B cells.

Figure S3. Absence of CSR following specific stimulations

(A-C) Representative FACS plots of activated CH12 cells and random Iα-deleted clones. CH12 line and Iα-deleted clones fail to switch to IgG3 upon LPS stimulation (**A**), to IgG2a upon LPS+IFNγ stimulation (**B**), or to IgG1 upon LPS+IL4 stimulation (**C**). At day 4 post-stimulation, the cells were stained with the indicated antibodies and analyzed by FACS. (UNS, unstimulated).

Figure S4. Post-switch transcripts following specific stimulations

(**A-C**) RT-qPCR quantification of post-switch transcripts (PSTs) levels (day 4) in activated splenic B cells, CH12 cells, and clones 5, 6 and 8 in response to LPS (Iµ-Cγ3) (**A**), to LPS+IFNγ (Iµ-Cγ2a) (**B**), and to LPS+IL4 (Iµ-Cγ1 and Iµ-Cε) (**C**) (n=3).

Figure S5. Switch transcription in unstimulated CH12 and deletion clones

RT-qPCR quantification of Sx pre-switch transcripts levels in unstimulated CH12 cells and Iα-deleted clones 3, 5 and 8. *Actin* transcripts were used for normalization (n≥3).

Figure S6. Iµ-Cγ2b post-switch transcripts following LPS and LIT stimulations

(A, B). RT-qPCR quantification of Iµ-Cγ2b post-switch transcripts (PSTs) levels (day 4 post-stimulation) in clones 5, 6 and 8 in response to LPS (**A**) or to LIT (**B**). **(C)**

Comparison of Iµ-Cγ2b post-switch transcripts levels in Iα-deleted clones 5, 6 and 8, following LPS and LIT stimulation (n=3).

Figure S7. Sε transcription following LPS+IL4 (LI) and CIT stimulation

RT-qPCR quantification of Sε pre-switch transcripts levels in CH12 cells and Iα-deleted clones 3, 5 and 8, activated with LPS+IL4 (LI) or CIT. *Actin* transcripts were used for normalization (n≥3).

Fig. S1

A

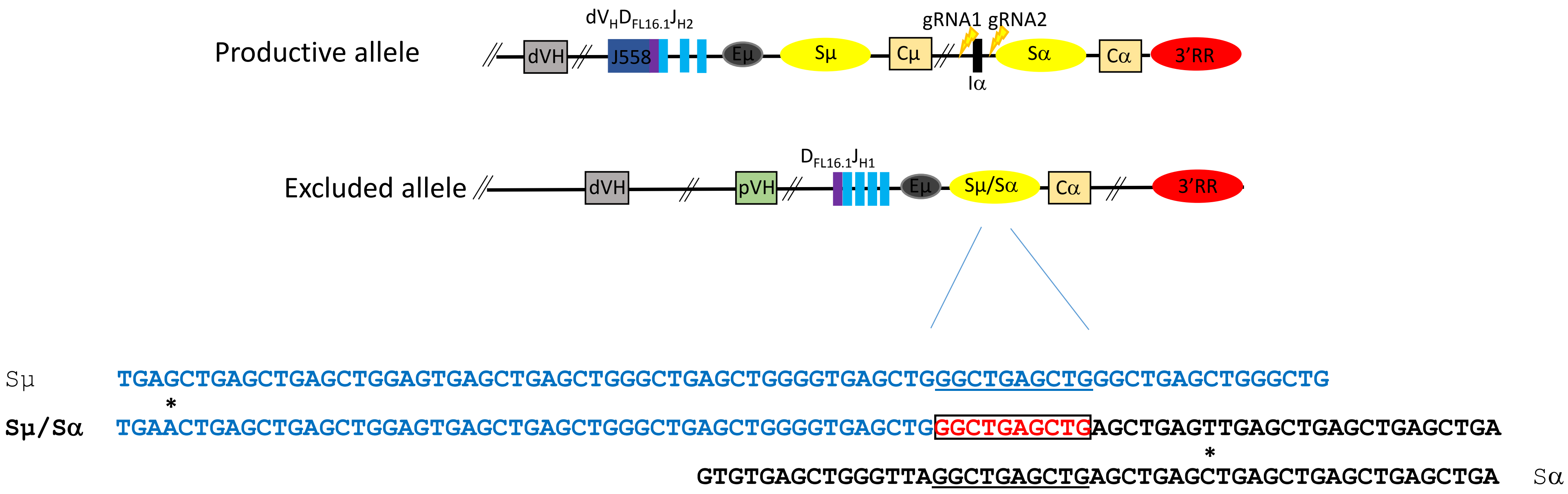

Productive allele
dVH
J558
$dV_HD_{FL16.1}J_{H2}$
Eμ
Sμ
Cμ
gRNA1 gRNA2
Iα
Sα
Cα
3’RR
Excluded allele
dVH
pVH
$D_{FL16.1}J_{H1}$
Eμ
Sμ/Sα
Cα
3’RR
Sμ TGAGCTGAGCTGAGCTGGAGTGAGCTGAGCTGGGCTGAGCTGGGGTGAGCTGGGCTGAGCTGGGCTGAGCTGGGCTG
*
Sμ/Sα TGAACTGAGCTGAGCTGGAGTGAGCTGAGCTGGGCTGAGCTGGGGTGAGCTGGGCTGAGCTGAGCTGAGTTGAGCTGAGCTGAGCTGA
*
GTGTGAGCTGGGTTAGGCTGAGCTGAGCTGAGCTGAGCTGAGCTGAGCTGA Sα

Fig. S1

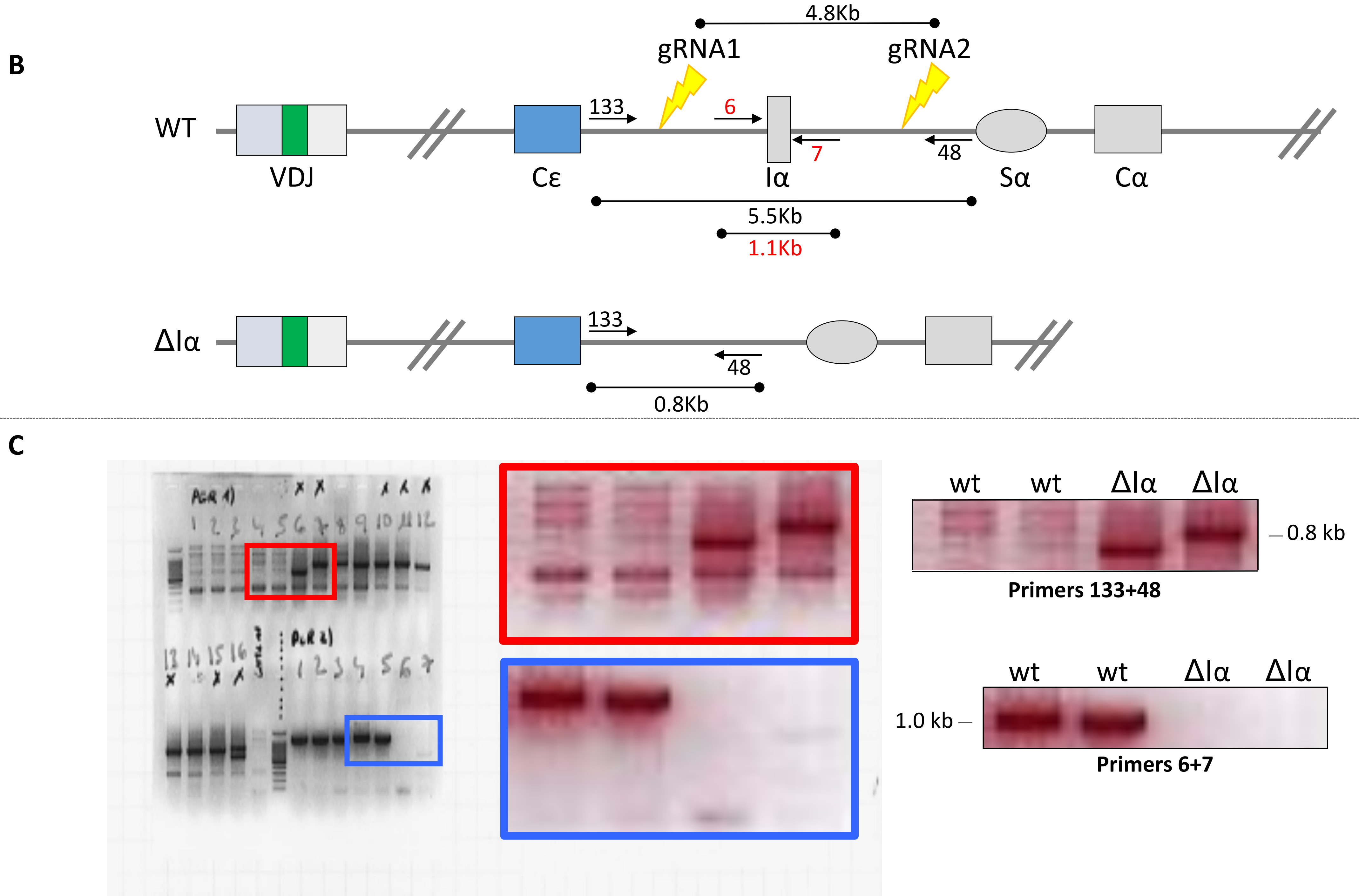
B
4.8Kb
gRNA1
gRNA2
WT
133
6
7
48
VDJ
Cε
Iα
Sα
Cα
5.5Kb
1.1Kb
ΔIα
133
48
0.8Kb
C
wt
wt
ΔIα
ΔIα
0.8 kb
Primers 133+48
wt
wt
ΔIα
ΔIα
1.0 kb
Primers 6+7

Fig. S1

D

```
ΔIα-1:  GCTCAGCACC GTGGAGAAAC TAAGAGATAA GC------  TG GGCTAATCCA AGCTAGGTTG  CCTGAGCTGG GCTAGGCTGA GCTGAGCTAG GCTGGAATAG GCTGGGCTAG GCTCATGTGA
ΔIα-2:  GCTCAGCACC GTGGAGAAAC TAAGAGATAA GCCG--  ACTG GGCTAATCCA AGCTAGGTTG  CCTGAGCTGG GCTAGGCTGA GCTGAGCTAG GCTGGAATAG GCTGGGCTAG GCTCATGTGA
ΔIα-3:  GCTCAGCACC GTGGAG---- ---------- ------------ ---------- --CTAGGTTG  CCTGAGCTGG GCTAGGCTGA GTTGAGTTAG GCTGGAATAG GCTGGGCTAG GCTCATGTGA
ΔIα-4:  GCTCAGCACC GTGGAGAAAC TAAGAGATAA GCCG  TAACTG GGCTAATCCA AGCTAGGTTG  CCTGAGCTGG GCTAGGCTGA GCTGAGCTAG GCTGGAATAG GCTGGGCTAG GCTCATGTGA
ΔIα-5:  --TCAATAAC TGGG------ ---------- ------------ --CTAATCCA AGCTAGGTTG  CCTGAGCTGG GCTAGGCTGA GCTGAGTTAG GCTGGAATAG GCTGGGCTAG GCTCATGTGA
ΔIα-6:  GCTCAGCACC GTGGAGAAAC TAAGAGATAA GCCG  TAACTG GGCTAATCCA AGCTAGGTTG  CCTGAGCTGG GCTAGGCTGA GCTGAGCTAG GCTGGAATAG GCTGGGCTAG GCTCATGTGA
ΔIα-7:  GCTCAGCACC GTGGAGAAAC TAAGAGATAA GCCG-  AACTG GGCTAATCCA AGCTAGGTTG  CCTGAGCTGG GCTAGGCTGA GCTGAGCTAG GCTGGAATAG GCTGGGCTAG GCTCATGTGA
ΔIα-8:  GTGGAGAAAC TGGGCTAGGG TT G GATGG GCTCAATAACTG GGCTAATCCA AGCTAGGTTG  CCTGAGCTGG GCTAGGCTGA GCTGAGCTAG GCTGGAATAG GCTGGGCTAG GCTCATGTGA
```

E

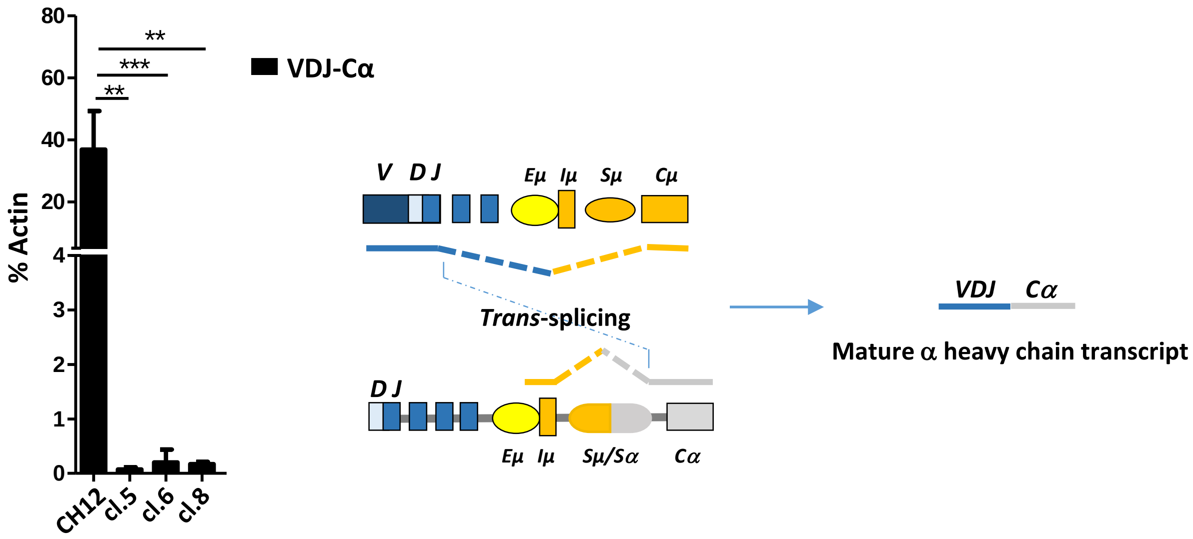

Fig. S2

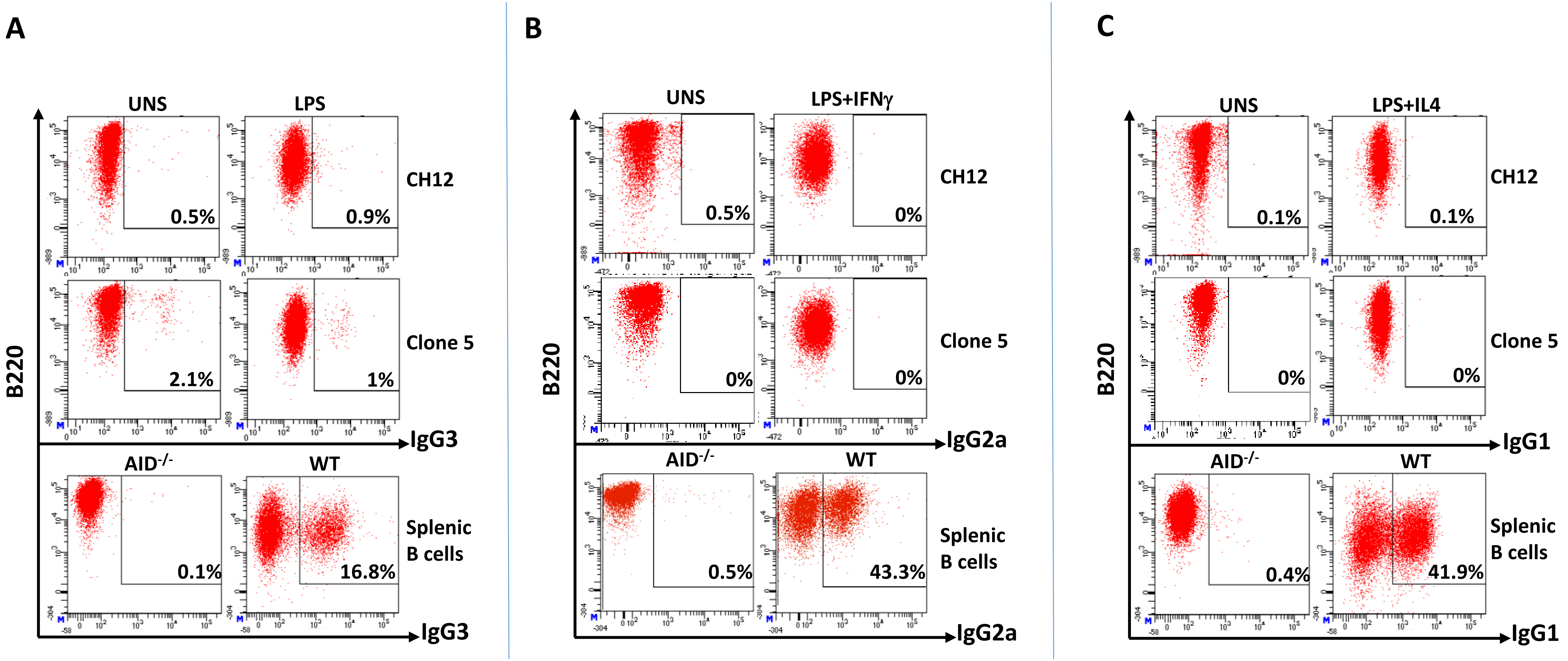
A
UNS
LPS
CH12
0.5%
0.9%
Clone 5
2.1%
1%
B220
IgG3
AID-/-
WT
Splenic B cells
0.1%
16.8%
IgG3
B
UNS
LPS+IFNγ
CH12
0.5%
0%
Clone 5
0%
0%
B220
IgG2a
AID-/-
WT
Splenic B cells
0.5%
43.3%
IgG2a
C
UNS
LPS+IL4
CH12
0.1%
0.1%
Clone 5
0%
0%
B220
IgG1
AID-/-
WT
Splenic B cells
0.4%
41.9%
IgG1

Fig. S3

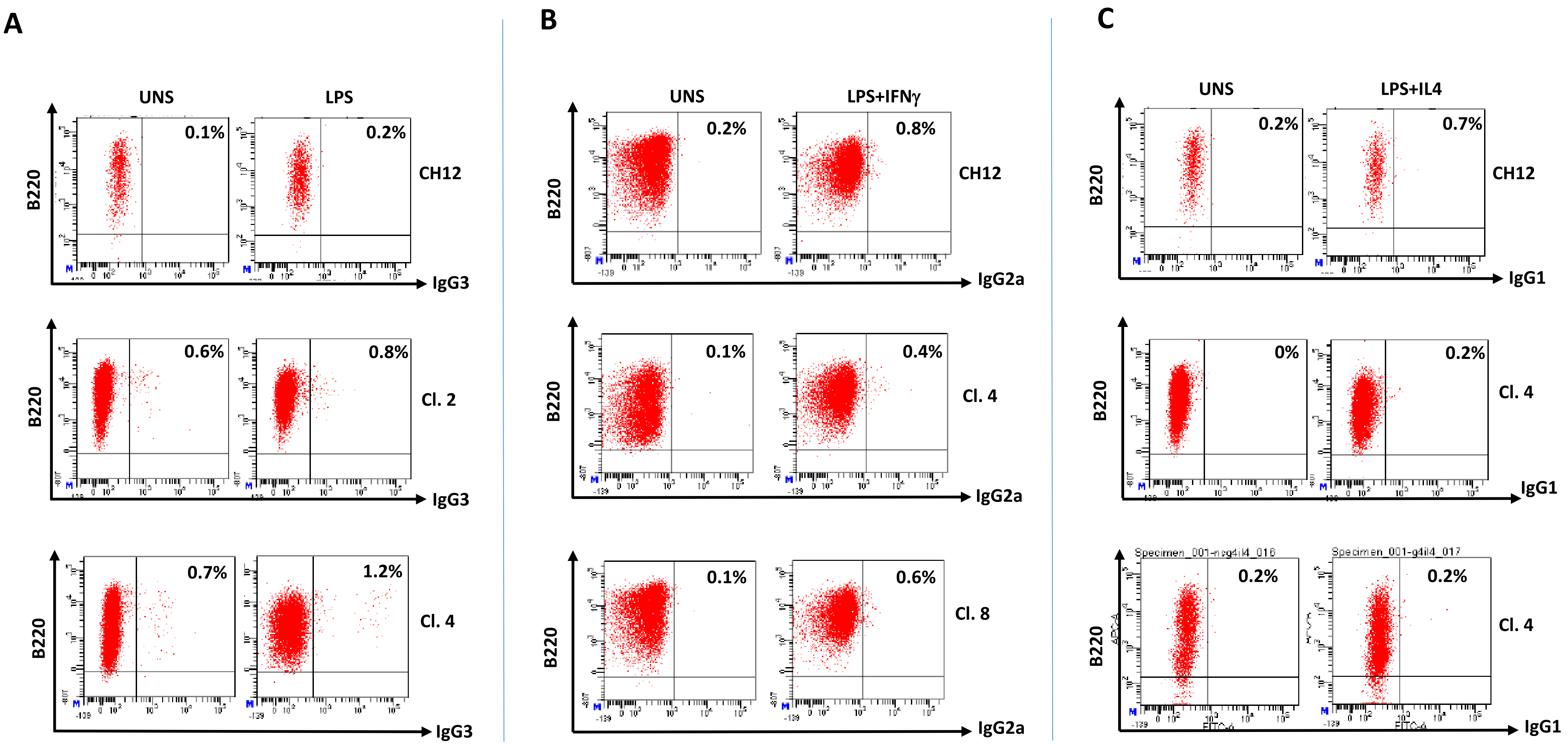
A
UNS
LPS
B220
IgG3
CH12
Cl. 2
Cl. 4
0.1%
0.2%
0.6%
0.8%
0.7%
1.2%
B
UNS
LPS+IFNγ
B220
IgG2a
CH12
Cl. 4
Cl. 8
0.2%
0.8%
0.1%
0.4%
0.1%
0.6%
C
UNS
LPS+IL4
B220
IgG1
CH12
Cl. 4
Cl. 4
0.2%
0.7%
0%
0.2%
0.2%
0.2%
Specimen_001-neg4il4_016
Specimen_001-g4il4_017

Fig. S4

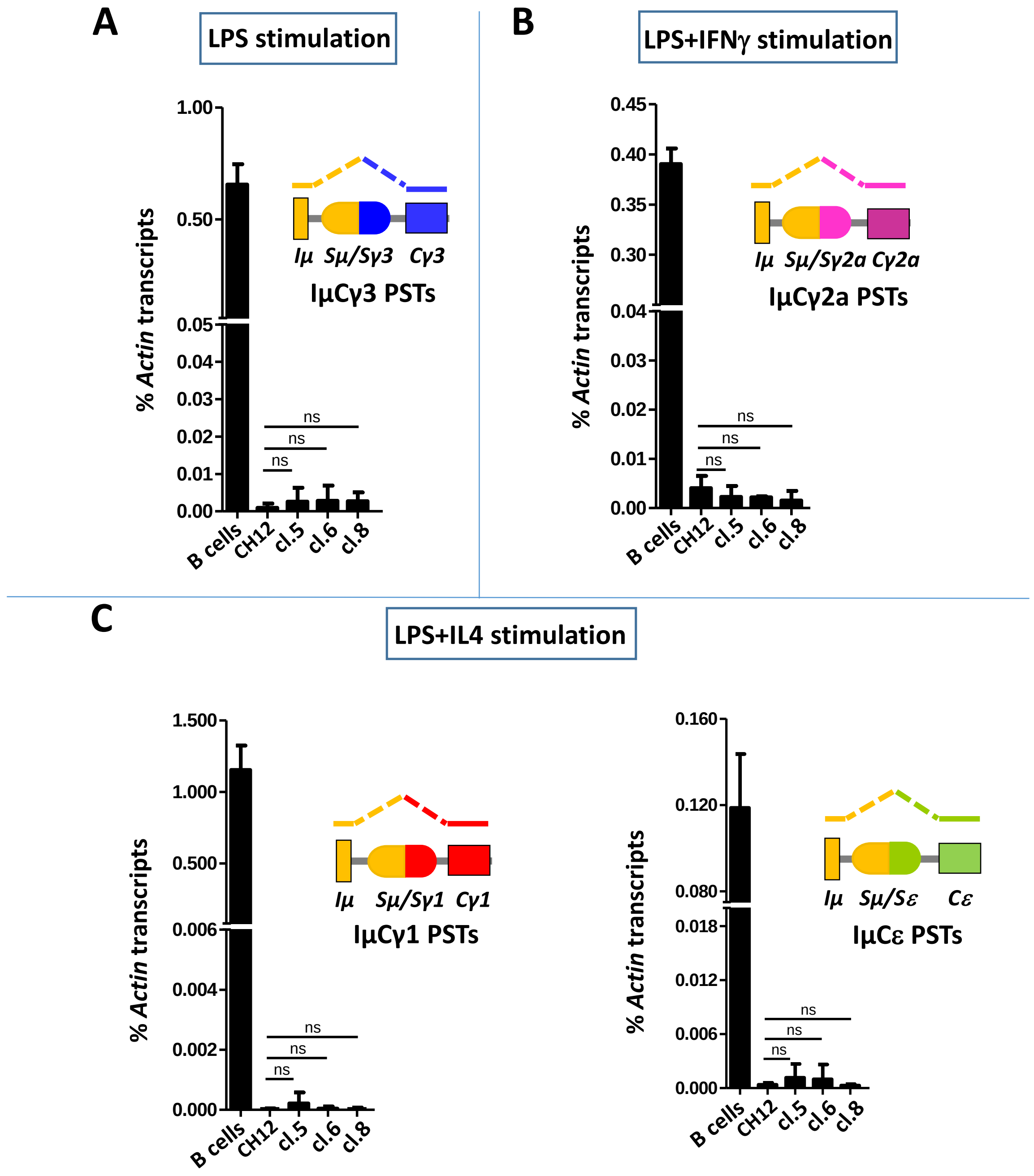

A
LPS stimulation
% Actin transcripts
1.00
0.50
0.05
0.04
0.03
0.02
0.01
0.00
ns
ns
ns
Iμ Sμ/Sγ3 Cγ3
IμCγ3 PSTs
B cells
CH12
cl.5
cl.6
cl.8
B
LPS+IFNγ stimulation
% Actin transcripts
0.45
0.40
0.35
0.30
0.04
0.03
0.02
0.01
0.00
ns
ns
ns
Iμ Sμ/Sγ2a Cγ2a
IμCγ2a PSTs
B cells
CH12
cl.5
cl.6
cl.8
C
LPS+IL4 stimulation
% Actin transcripts
1.500
1.000
0.500
0.006
0.004
0.002
0.000
ns
ns
ns
Iμ Sμ/Sγ1 Cγ1
IμCγ1 PSTs
B cells
CH12
cl.5
cl.6
cl.8
% Actin transcripts
0.160
0.120
0.080
0.018
0.012
0.006
0.000
ns
ns
ns
Iμ Sμ/Sε Cε
IμCε PSTs
B cells
CH12
cl.5
cl.6
cl.8

Fig. S5

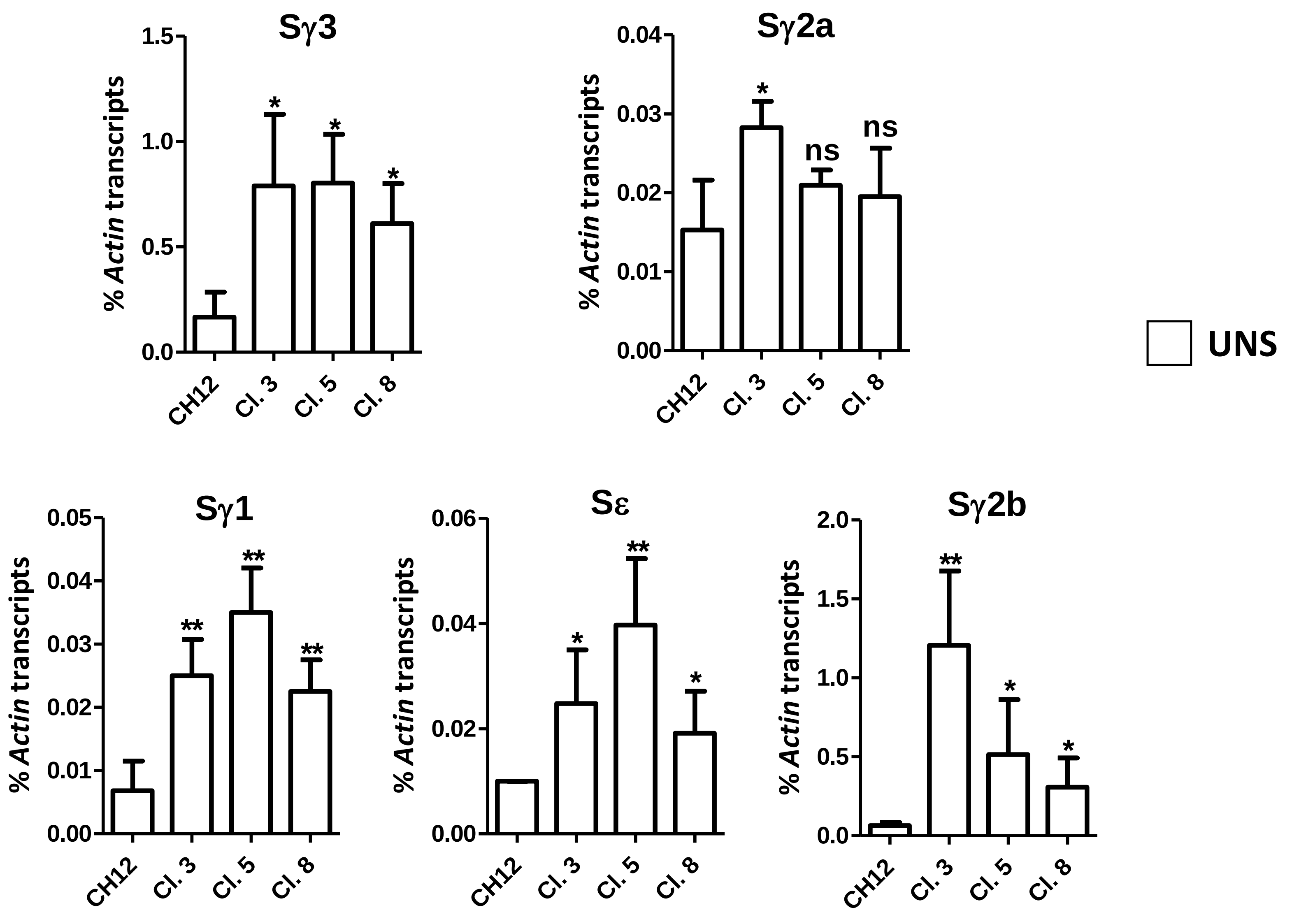

Sγ3
% Actin transcripts
1.5
1.0
0.5
0.0
*
*
*
CH12
Cl. 3
Cl. 5
Cl. 8
Sγ2a
% Actin transcripts
0.04
0.03
0.02
0.01
0.00
*
ns
ns
CH12
Cl. 3
Cl. 5
Cl. 8
UNS
Sγ1
% Actin transcripts
0.05
0.04
0.03
0.02
0.01
0.00
**
**
**
CH12
Cl. 3
Cl. 5
Cl. 8
Sε
% Actin transcripts
0.06
0.04
0.02
0.00
*
**
*
CH12
Cl. 3
Cl. 5
Cl. 8
Sγ2b
% Actin transcripts
2.0
1.5
1.0
0.5
0.0
**
*
*
CH12
Cl. 3
Cl. 5
Cl. 8

Fig. S6

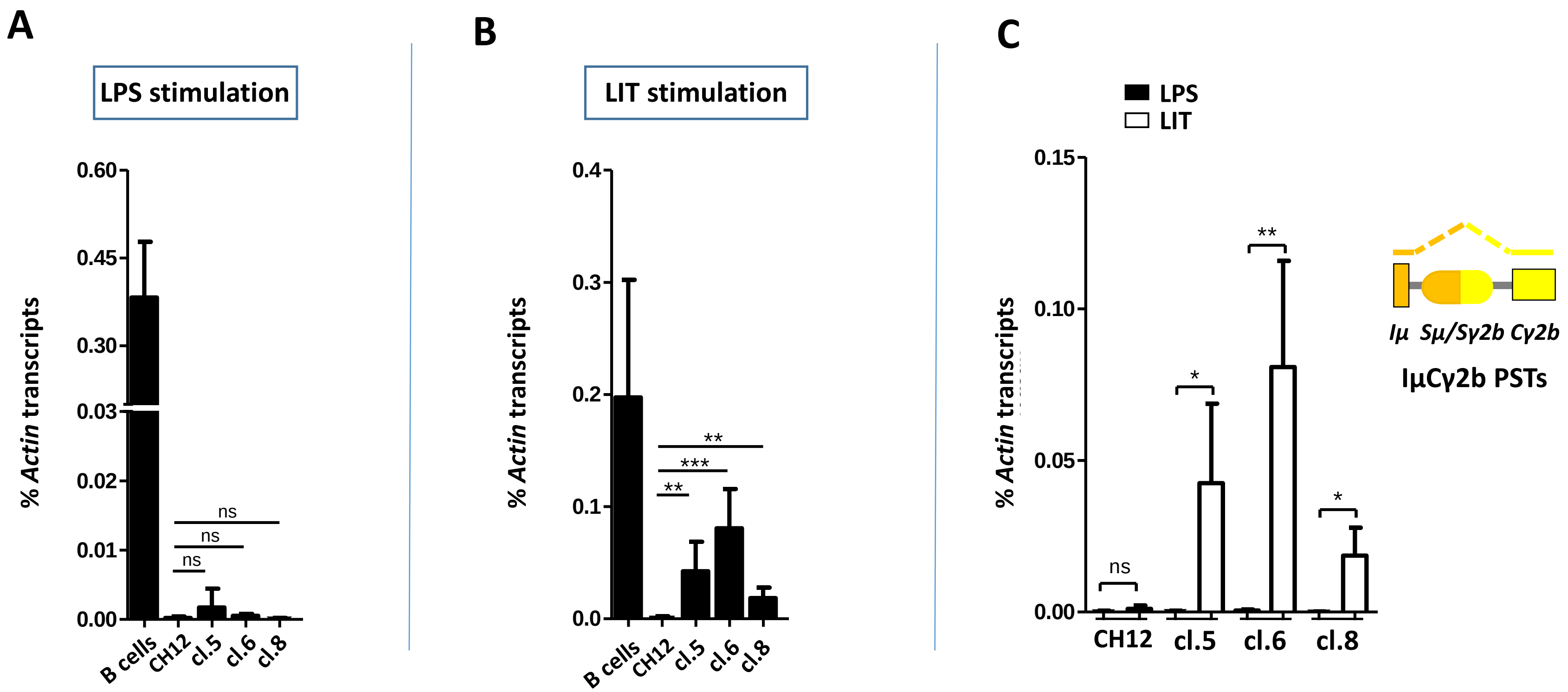

A
LPS stimulation
% Actin transcripts
0.60
0.45
0.30
0.03
0.02
0.01
0.00
ns
ns
ns
B cells
CH12
cl.5
cl.6
cl.8
B
LIT stimulation
% Actin transcripts
0.4
0.3
0.2
0.1
0.0
**
***
**
B cells
CH12
cl.5
cl.6
cl.8
C
LPS
LIT
% Actin transcripts
0.15
0.10
0.05
0.00
ns
*
**
*
CH12
cl.5
cl.6
cl.8
Iμ Sμ/Sγ2b Cγ2b
IμCγ2b PSTs

Fig. S7

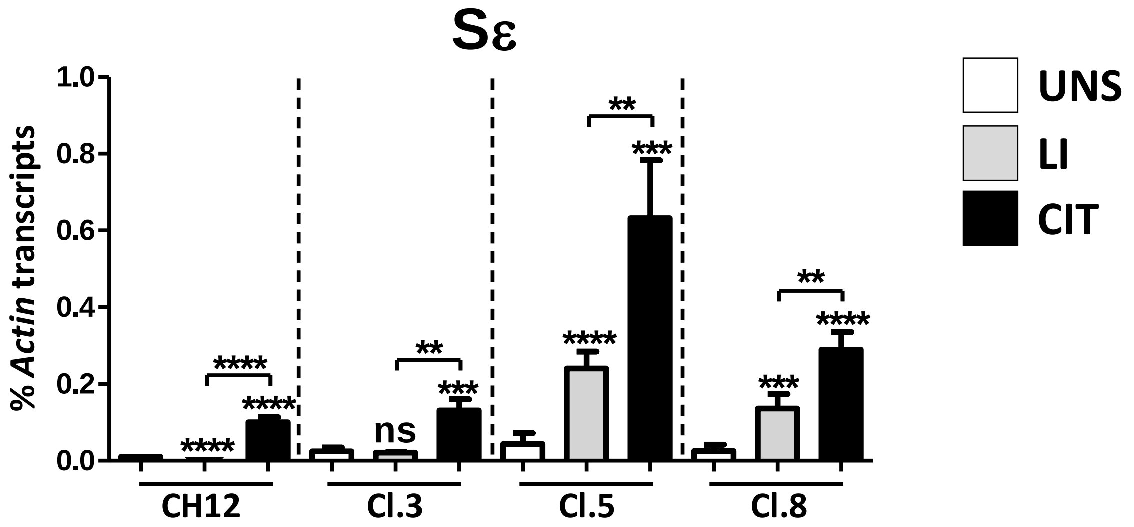

Sε
% *Actin* transcripts
1.0
0.8
0.6
0.4
0.2
0.0
****
****
****
ns
**
***
**
***
****
**
****
***
CH12
Cl.3
Cl.5
Cl.8
UNS
LI
CIT

Table S1

Primers used in this study

Primer	Sequence
CRISPR/Cas9-mediated deletion of Iα promoter/exon	
**Guide RNAs**	
**gRNA1-fw**	caccgAACTAAGAGATAAGCCGCCC
**gRNA1-rev**	aaaccGGGCGGCTTATCTCTTAGTT
**gRNA2-fw**	caccGGTTGGATGGGCTCAATAAC
**gRNA2-rev**	aaacGTTATTGAGCCCATCCAACC
**Cloning primers**	
**gRNA-MluIFw**	acgcgtGAATGGCGCATGTGAGGGC
**gRNA-MluIRev**	acgcgtGTAACGGGTACCCCATTTGTCTGC
**PCR Screening**	
**Primer 6**	gccactagtTCCCTTCTACTCTTAATTGTCCTCTCT
**Primer 7**	CTAGTTCAGTTCATCCCATTCCAGAC
**Primer 48**	GCCTAACCCAGCTAACACTAGTC
**Primer 133**	GAACTCACATGACAAAAGACAGGC
Transcription Analysis	
**Switch Transcription**	
**Iγ3-Fw**	TGGGCAAGTGGATCTGAACAC
**Cγ3-Rev**	CTCAGGGAAGTAGCCTTTGACA
**Iγ1-Fw**	GGCCCTTCCAGATCTTTGAG
**Cγ1-Rev**	GGATCCAGAGTTCCAGGTCAC
**Iγ2b-Fw**	CACTGGGCCTTTCCAGAACTA
**Cγ2b-Rev**	CACTGAGCTGCTCATAGTGTA
**Iγ2a-Fw3**	GAAGGTTCATCGGGAAAGGC
**Cγ2a-Rev1**	GCCAGTTGTATCTCCACACACAG
**Iε-Fw3**	ACTAGAGATTCACAACGCCTGGGA
**Cε-R3**	AGGGTCATGGAAGCAGTGCCTTTA

**Iα-ST-Fw**	GGGTGACTCAGGCTGTTGTGG
**CαR**	GAGCTGGTGGGAGTGTCAGTG
***Aicda* transcription**	
**AID Fw**	GGAGACCGATATGGACAGCC
**AID Rev**	AGAGGTAGGTCTCATGCCGT
**3'RR eRNAs**	
**HS3a-F**	GGCTCCTGTACTAGATCGATGG
**HS3a-R**	ACTGTCCCAGTTGCAGCCC
**HS1.2-3'F**	GGGTGGCTCAACACCCCAGG
**HS1.2-3'R**	TGGGCTGAGGCAGGCCAAGA
**HS3b-F1**	TGAGGGCCAGGGCCCAATGA
**HS3b-R1**	GGATCTCGGTCCTGGTAACTGGCT
***Trans*-splicing**	
**VHJ558-Fw**	GCGAAGCTTARGCCTGGGRCTTCAGTGAAG
**CαR**	GAGCTGGTGGGAGTGTCAGTG
**Normalization**	
**Actin 4-Fw**	TACCTCATGAAGATCCTGA
**Actin 5-Rev**	TTCATGGATGCCACAGGAT
